# Self-Criticism in Preventive Guided Self-Help Interventions: Greater Gains or Greater Risks? Its Effect on Adherence, Treatment Success, and Working Alliance

**DOI:** 10.3390/healthcare14081107

**Published:** 2026-04-21

**Authors:** Micaela Di Consiglio, Francesca D’Olimpio, Alessandro Couyoumdjian

**Affiliations:** 1 Department of Psychology, Faculty of Medicine and Psychology, Sapienza University of Rome, 00185 Rome, Italy; alessandro.couyoumdjian@uniroma1.it; 2 Department of Psychology, University of Campania Luigi Vanvitelli, 81100 Caserta, Italy; francesca.dolimpio@unicampania.it

**Keywords:** self-criticism, guided self-help intervention, Internet, prevention strategies, mental health, university students

## Abstract

**Highlights:**

**What are the main findings?**

- Participants with severe self-criticism showed the largest improvements in emotional and cognitive processes targeted by the intervention.
- Participants with moderate levels of internalized and comparative self-criticism were more likely to drop out and showed lower adherence to the program.

**What are the implications of the main findings?**

- In preventive and low-intensity interventions, self-criticism may function as a catalyst for change rather than a barrier to engagement.
- Digital preventive programs may benefit from tailoring strategies to support individuals with moderate levels of self-criticism better to reduce dropout risk.

**Abstract:**

Background: Self-criticism is a transdiagnostic factor associated with psychological distress and poorer outcomes in traditional psychotherapy, yet recent evidence suggests it may facilitate change in preventive and low-intensity interventions. This study examined the role of self-criticism in adherence, working alliance, and outcomes within NoiBene, a guided self-help program designed to promote well-being and prevent psychological distress among non-clinical university students. Methods: A total of 455 participants (82% female; M = 23.5 years) completed measures of internalized and comparative self-criticism, and key psychological processes (e.g., emotional awareness, rumination, worry, perfectionism, psychological inflexibility, and assertiveness) were assessed before and after the intervention. Adherence and working alliance were measured only after the intervention. Results: Contrary to evidence from clinical settings, severe self-criticism was not associated with increased dropout or weaker alliance. Instead, individuals with severe self-criticism exhibited the greatest improvements across multiple domains, suggesting a higher potential for therapeutic gain. Moreover, participants with moderate levels of both internalized and comparative self-criticism showed higher dropout and lower adherence. Conclusions: These findings indicate that, in preventive guided self-help contexts, self-criticism does not necessarily hinder engagement and outcomes and may, under certain conditions, function as a catalyst for change. Implications for tailoring digital preventive interventions and addressing dropout risk are discussed.

## 1. Introduction

Self-criticism, a process of negative self-evaluation, manifests as a critical, self-blaming internal dialog that can lead to a sense of personal inadequacy and feelings of self-hatred and self-disgust [1]. Self-criticism, especially in its more severe and pervasive forms, is considered a transdiagnostic psychopathological factor, as it contributes to the onset and maintenance of several mental disorders, such as depression, anxiety disorders, eating disorders, and post-traumatic stress disorder [2]. Self-criticism can also hinder goal progress [3] and is associated with interpersonal problems, low self-esteem, feelings of guilt and shame, and a sense of chronic failure [2].

Evidence from clinical settings suggests that self-criticism also influences the therapeutic process. First, patients with severe self-criticism have greater difficulty forming and maintaining a therapeutic alliance [4], probably due to the interpersonal problems commonly linked with self-criticism, such as high interpersonal sensitivity and fear of judgment [5,6]. Therefore, these individuals may find it harder to trust the therapist and make less disclosure during treatment, which can ultimately compromise the therapeutic alliance [7]. Second, severe self-criticism is associated with poorer outcomes, as indicated by a recent meta-analysis suggesting that baseline levels of self-criticism at the beginning of the therapy predict poorer therapy outcomes across various treatments and diagnoses [8]. Lastly, it has been suggested that baseline levels of self-criticism predict a higher probability of dropping out of therapy, even when controlling for therapeutic alliance [9].

While findings from clinical contexts suggest a negative influence of self-criticism, recent evidence indicates that, under the right conditions, self-criticism also confers greater capacity for improvement. In particular, across preventive and low-intensity interventions, such as Internet-based self-help and guided self-help interventions [10], there is evidence that baseline self-criticism can predict greater outcomes in subclinical samples [11,12,13]. For example, a study examining the effects of positive psychology interventions found that self-criticism predicted greater reductions in depression and increases in life satisfaction for up to six months [12]. Similarly, in a mindfulness-based program, those with a greater decrease in depressed mood were those with higher baseline self-criticism levels [11], while, in a Compassion-Focused Training, participants with higher baseline self-criticism exhibited greater reductions in anxiety and depression, as well as more significant improvements in self-compassion and positive affect, for up to three months [13]. The differences observed in the role of self-criticism between psychotherapy and preventive interventions do not appear to stem from methodological or measurement factors. Both types of studies employed longitudinal designs and used similar, well-validated instruments to assess self-criticism. Instead, these differences may be better explained by variations in participants’ symptom severity. Studies in psychotherapy contexts primarily involved clinical populations with diagnosed mental disorders [8]. In contrast, low-intensity intervention studies included more heterogeneous samples: some focused on individuals with mild to moderate symptoms [12], others involved both clinical and non-clinical participants [11], and some did not report symptom severity at all [13]. Given that higher levels of self-criticism are associated with more severe psychological distress [14], it is plausible, although not explicitly stated in most studies, that baseline levels of self-criticism also differed across samples.

### The Present Study

Low-intensity and preventive interventions often target highly heterogeneous populations. These programs may be delivered universally to entire groups regardless of individual risk, targeted to those at elevated risk, or selectively offered to individuals with subclinical symptoms [15]. As such, individuals participating in these interventions may exhibit a wide range of self-criticism. Investigating the role of self-criticism in these contexts is therefore essential, as it can clarify for whom preventive interventions are most effective, under what conditions, and whether self-criticism acts as a barrier to or facilitator of change. Understanding how self-criticism influences therapeutic engagement and outcomes can also inform the development of tailored interventions and guide adaptations that improve accessibility and impact for those most at risk. Therefore, the present study aimed to examine the role of self-criticism in influencing key therapeutic processes, including adherence, working alliance, and intervention outcomes within NoiBene, a guided self-help intervention designed to promote psychological well-being and prevent psychological distress among nonclinical university students [16]. Grounded in cognitive-behavioral therapy and positive psychology principles, NoiBene is a multi-component program designed to promote a series of skills associated with well-being and self-realization, such as emotional awareness, assertiveness and goal-setting, and to reduce psychological processes that are implicated in the onset and maintenance of mental health difficulties, such as perfectionism, self-criticism, rumination, worry and psychological inflexibility. NoiBene consists of a series of online modules, each dedicated to a specific topic, and each module combines psychoeducation, experiential exercises, guided self-reflection, and self-monitoring tools. In addition, NoiBene incorporates regular meetings with a designated guide (the “Tutor”), whose role is to support participants in engaging consistently with the program. These sessions are intended to reduce dropout rates, sustain motivation and adherence, and provide an opportunity to review completed activities, clarify complex concepts, and address any difficulties encountered during the intervention.

Given the multiple conceptualizations of self-criticism [1], the present study adopts the two-dimensional model that distinguishes internalized self-criticism (i.e., negative self-evaluation based on unrealistically high personal standards) from comparative self-criticism (i.e., feelings of inferiority toward others perceived as superior). Based on previous findings, we hypothesized that higher self-criticism would predict greater adherence and improvement after the intervention. Evidence from preventive and low-intensity programs suggests that, in non-clinical populations, self-criticism can motivate self-improvement rather than hinder it [11,12,13]. In this context, elevated self-criticism may indicate awareness of personal limitations and a stronger drive for change, unlike in clinical settings where it often leads to shame, hopelessness, and dropout [4,8]. We also expected that higher comparative self-criticism, due to its link with chronic self-doubt and negative social comparison [17], might weaken perceptions of the working alliance with the guide. The study was approved by the Ethical Board at Sapienza University of Rome and adhered to the principles outlined in the Declaration of Helsinki.

## 2. Materials and Methods

### 2.1. Participants

NoiBene was advertised on the university’s official channels, including the newsletter and website. NoiBene was presented as a guided self-help program designed to enhance life skills associated with well-being and self-realization, while reducing negative coping strategies that contribute to emotional suffering. The inclusion criteria were: being at least 18 years old, having a stable Internet connection, and having sufficient reading, understanding, and speaking skills in Italian. Based on the stepped-care model [18], NoiBene is an intervention addressed to individuals with no or mild symptoms, and it is not appropriate for treating mental disorders. For this reason, only participants who declare not having a diagnosis of a mental disorder were included in the study.

After removing the outliers, 455 students were included in the study. Most participants were female (F = 82.4%; n = 375) with a minority of male (M = 17.6%; n = 80). The mean age of the sample is 23.5 (SD = 2.90), ranging from 19 to 41 years. Most of the sample consists of students enrolled in a bachelor’s degree program (49.9%; n = 227), followed by those in a master’s degree program (44.4%; n = 202), a five-year single program (4.4%; n = 20), or a Ph.D. program (1.3%; n = 6). Most students are enrolled in a human or social science course (42.4%; n = 193) and a psychology course (37.1%; n = 169). Other students attend courses related to mathematics, physics, and natural sciences (9.2% n = 42), helping professionals (6.2%; n = 28), architecture or engineering (2.9%; n = 13), and educational science (2.2%; n = 10).

### 2.2. Measures

#### 2.2.1. Socio-Demographic and Motivational Questionnaire

An ad hoc questionnaire was proposed to collect information on socio-demographic variables (e.g., age, gender, occupational status, university status) and motivations for participation (e.g., gaining a better understanding of themselves, improving themselves, addressing psychological problems they experience).

#### 2.2.2. Mental Well-Being

The Warwick–Edinburgh Mental Well-being Scale (WEMWBS) [19,20] is a 14-item questionnaire on a 5-point scale that measures positive feelings and functioning, encompassing both hedonic and eudaimonic aspects of well-being. It provides a single mental well-being score, with higher scores suggesting higher mental well-being. The Italian version comprises 12 items and has a Cronbach’s alpha of 0.86. In our sample, the Cronbach alpha was 0.90.

#### 2.2.3. Emotional Awareness

The Toronto Alexithymia Scale (TAS-20) [21,22] was used to assess the ability to identify and communicate feelings, a skill related to emotional awareness. The scale, composed of 20 items on a 5-point Likert scale, measures the two constructs in terms of difficulty; therefore, a higher score indicates greater difficulty in identifying and communicating feelings. In the Italian version, Cronbach’s alpha is 0.77 for the first dimension and 0.67 for the second. In our sample, Cronbach’s alpha was 0.81 for the difficulty identifying feelings dimension and 0.82 for the difficulty communicating feelings dimension.

#### 2.2.4. Perfectionism

The Multidimensional Perfectionism Scale (MPS) [23,24] is a 35-item questionnaire that measures five dimensions of perfectionism: personal standards, concern over mistakes, parental expectations, doubting actions, and organization. In the Italian version, the dimensions “concern over mistakes” and “doubting of actions” are incorporated into a single dimension. For this study, the only dimensions used were personal standards (PS) and concern over mistakes and doubting of actions (CMD). All items are scored on a 5-point Likert scale. Higher scores indicate greater perfectionism. In the Italian version, the Cronbach alpha for the two dimensions is 0.76 and 0.86, respectively. In our sample, the Cronbach alpha was 0.81 for the first dimension and 0.91 for the second.

#### 2.2.5. Self-Criticism

The Level of Self-criticism Scale (LOSC) [17,25] is a 22-item questionnaire measuring two dimensions of self-criticism: internalized and comparative self-criticism. Internalized self-criticism (ISC) is defined as a negative view of oneself in comparison to personal standards, whereas comparative self-criticism (CSC) involves judging oneself negatively in comparison to others, who are perceived as better, more capable, and often more critical. The responses are given on a 7-point Likert, with higher scores indicating higher self-criticism. In the Italian version, internal consistency for the two dimensions was good (ISC α = 0.87; CSC α = 0.81). In our sample, the Cronbach alpha was 0.90 for internalized self-criticism and 0.79 for comparative self-criticism.

#### 2.2.6. Repetitive Thinking

The Ruminative Response Scale (RRS) [26,27] and the Worry Penn State Questionnaire (PSWQ) [28,29] were used to measure rumination and worry, respectively. The RRS is a 22-item questionnaire that assesses how often individuals ruminate when feeling down, sad, or depressed, using a 4-point Likert scale. Higher scores indicate higher tendency to ruminate. The Cronbach’s alpha in the Italian version was good (α = 0.80), as was the value in our sample (α = 0.90). The PSWQ is a 16-item self-report instrument using a 5-point Likert scale to measure the tendency to worry, regardless of the content of the worry. Higher scores indicate higher tendency to worry. The Cronbach alpha values in the Italian version (α = 0.85) and in our sample (α = 0.94) were good.

#### 2.2.7. Experiential Avoidance

The Acceptance and Action Questionnaire-II (AAQ-II) [30,31] is a 10-item questionnaire on a 7-point Likert scale measuring psychological inflexibility, a cornerstone of experiential avoidance. The items focus on the inability to remain open to thoughts, feelings, and other internal experiences while pursuing meaningful actions. Higher scores indicate greater psychological inflexibility. The Cronbach alpha of the Italian version was 0.83, whereas in our sample it was 0.89.

#### 2.2.8. Assertiveness

The Scale for Interpersonal Behavior (SIB) [32,33] is a 50-item questionnaire that assesses assertiveness. Each item is presented twice: first, participants indicate the level of discomfort the described situation would cause them; second, they report how frequently they engage in the behavior, regardless of any discomfort. Each item requires two separate responses using 5-point Likert scales. The items are categorized into four dimensions: negative assertion (e.g., the ability to assert one’s rights, say no, and express negative emotions), personal limitation (e.g., the capacity to acknowledge one’s limitations, apologize, and ask for help), initiating assertiveness (e.g., the ability to begin or end conversations, share opinions, and express disagreement), positive assertion (e.g., the ability to give and receive compliments, show appreciation, and express affection). Moreover, the scale provides an overall measure of assertiveness. For each dimension, two scores are generated: one reflecting discomfort and the other representing the frequency of the behavior. For the discomfort dimension, higher scores suggest increased distress during communication. For the frequency dimension, higher scores represent a higher rate of communicative behavior, independent of the associated discomfort. For the purpose of the study, online the overall measures of assertiveness were used. In the Italian version, Cronbach’s alphas range from 0.78 to 0.83 for both the discomfort and frequency scales, covering the four dimensions of each scale. The Cronbach’s alpha for the general score, in both scales, is 0.93. In the present study, Cronbach’s alphas for the four discomfort scales ranged from 0.82 to 0.90, and for the frequency scale from 0.84 to 0.89. Cronbach’s alpha for the general assertiveness scores was 0.95 in both scales.

#### 2.2.9. Working Alliance

The Working Alliance Inventory for Internet Interventions (WAI-I) [34] is a 12-item questionnaire on a 5-point Likert scale that assesses the therapeutic alliance in guided self-help online interventions. The questionnaire is composed of two subscales: (1) goal and task (e.g., agreement on the goal and the task); (2) bond (e.g., development of a collaborative bond). The Cronbach’s alphas are 0.93 and 0.89 for the two scales. For the present study, the original scale was translated into Italian through a translation-back-translation process. In the present study, Cronbach’s alphas are 0.84 and 0.82.

### 2.3. Procedure

Students interested in participating in the NoiBene intervention submitted a request via email. Upon signing the institutional informed consent form, which detailed study and privacy information in accordance with the General Data Protection Regulation (EU) 2016/679 (GDPR), each participant received a personal username and password to access their dedicated page on the NoiBene website. On this homepage, students first completed an ad hoc socio-demographic and motivation questionnaire. Once eligibility was confirmed based on predefined criteria, participants were presented with a series of standardized questionnaires assessing psychological well-being (WEMWBS), emotional awareness (TAS-20), perfectionism (MPS), self-criticism (LOSC), rumination (RRS), worry (PSWQ), psychological inflexibility (AAQ-II), and assertiveness (SIB). These measures were administered again at the end of the intervention to evaluate change over time, along with measures for adherence and working alliance (WAI-I). Following the assessment phase, participants began the NoiBene intervention. NoiBene was structured into a sequence of thematic modules, each focusing on a specific psychological skill (i.e., emotional awareness, assertiveness, goal setting) or transdiagnostic process (i.e., repetitive thinking, perfectionism, experiential avoidance). Participants were instructed to complete the modules independently and to engage with the different components of each module, including psychoeducational materials, experiential exercises, guided self-reflection activities, and self-monitoring tasks. Every two weeks, participants also attended meetings with a designated Tutor, aimed at supporting their engagement with the program. During these sessions, participants had the opportunity to review the content of the modules, discuss any difficulties encountered, clarify relevant concepts, and receive guidance to maintain motivation and adherence. Please refer to the Supplementary Materials (“1-NoiBene intervention”) for further information about the intervention.

### 2.4. Statistical Analysis

The analyses were run using Jamovi (version 2.6.44, The Jamovi project, Sydney, Australia). Descriptive analyses were conducted on all baseline data, and the assumptions of normality were assessed using skewness and kurtosis values. Baseline levels of self-criticism were used to categorize participants into mild, moderate, and severe groups based on tertile splits, employing a percentile rank method. The 33rd and 66th percentiles were used as thresholds: scores ≤ 33rd percentile were categorized as mild, scores between the 34th and 65th percentiles as medium, and scores ≥ 66th percentile as high self-criticism. This grouping was performed separately for internalized and comparative dimensions. One-way ANOVA and a Chi-square test were used to investigate differences in motivation, adherence, and dropout rates between participants with mild, moderate, and severe levels of self-criticism. Moreover, to examine changes in outcomes from pre- to post-intervention, a series of General Linear Models (GLMs) was conducted on the overall sample on WEMWBS, TAS-20, MPS, LOSC, RRS, PSWQ, AAQ-II, and SIB scores, while controlling for baseline levels of internalized and comparative self-criticism, as measured by the LOSC. For outcomes where a significant interaction with self-criticism emerged, further exploration was conducted. A series of repeated-measures ANOVAs was conducted to assess pre-post changes in all significant interaction effects that emerged in the previous analyses, with factors Time (Pre vs. Post) and Group (Mild vs. Moderate vs. Severe). Bonferroni post hoc analyses were run on the significant Time × Group interaction. Lastly, Pearson correlation analyses were conducted to examine the association between working alliance, as measured by the WAI-I, and self-criticism and between satisfaction with the intervention and self-criticism. Where significant correlations were found, one-way ANOVAs were used to investigate further group differences in working alliance and satisfaction across self-criticism levels. The level of significance was set at *p* < 0.05 for all analyses. Effect sizes were reported using partial eta squared (η^2^p) and interpreted according to Cohen’s guidelines: η^2^p = 0.01 (small), η^2^p = 0.06 (medium), and η^2^p = 0.14 (large). Missing data was handled appropriately, based on the nature and pattern of missingness.

## 3. Results

### 3.1. Preliminary Analyses

Normality assumptions were verified using skewness and kurtosis values. All variables fell within the acceptable range (skewness from −0.30 to 0.56; kurtosis from −0.93 to 0.21), indicating that the data were normally distributed and suitable for parametric analysis.

Baseline levels of self-criticism were used to categorize participants into mild, moderate, and severe groups based on tertile splits, employing a percentile rank method. Given that Internalized Self-Criticism (ISC) and Comparative Self-Criticism (CSC) are only partially associated with each other (r = 0.52, *p* < 0.001), separate groupings were performed for the internalized and comparative self-criticism dimensions, resulting in three groups for each (Table 1). The sizes of the resulting groups were not precisely equal despite the use of a tertile-based categorization. This slight variability is due to identical scores among participants, particularly near the percentile cut-off points. Nevertheless, this subdivision remains as faithful as possible to a proportional division of the sample into tertiles, accurately reflecting the empirical distribution of the data.

### 3.2. Motivation for Participation

A series of chi-square tests was conducted to examine whether participants’ motivations for enrolling in the NoiBene program varied as a function of their ISC and CSC levels. The motivations assessed included: (1) wanting to understand oneself better, (2) seeking personal improvement, and (3) addressing perceived psychological problems.

No significant associations were found between levels of ISC and any of the three motivations (Self-understanding: χ^2^(2) = 0.90, *p* = 0.64; Self-improvement: χ^2^(2) = 2.38, *p* = 0.30; Psychological problems: χ^2^(2) = 0.65, *p* = 0.72). Similarly, no significant associations were found between CSC and the motivations related to self-understanding (χ^2^(2) = 1.82, *p* = 0.40) or self-improvement (χ^2^(2) = 1.80, *p* = 0.41). A significant association emerged for the motivation related to psychological problems (χ^2^(2) = 7.05, *p* = 0.03). However, post hoc analysis using adjusted standardized residuals did not reveal any cell exceeding the conventional ±1.96 threshold for significance. The largest deviation was observed for participants reporting problems in the severe group (ASD = 1.91), indicating only a trend toward statistical significance.

### 3.3. Adherence and Dropout Rates

Adherence rates were measured by the modules that were effectively concluded. Of the eight modules in NoiBene, students completed an average of 7.53 (SD = 1.57). Most participants concluded the entire NoiBene intervention (90.8%; n = 413), whereas some dropped out at some point (9.2%; n = 42).

To examine the impact of ISC on adherence, a one-way ANOVA was conducted among participants with mild (n = 163), moderate (n = 150), and severe (n = 142) ISC. The analysis revealed a significant main effect of ISC on the number of completed modules, F(2, 452) = 14.12, *p* < 0.001. Bonferroni post hoc comparisons revealed that participants with moderate ISC completed significantly fewer modules (M = 6.99, SD = 2.29) than those with both mild (M = 7.81, SD = 0.93; *p* < 0.001) and severe (M = 7.79, SD = 0.94; *p* < 0.001) ISC. A chi-square test also revealed a significant association between ISC levels and dropout rates, χ^2^(2) = 17.91, *p* < 0.001. Adjusted residuals indicated that participants with moderate ISC were more likely to drop out (ASD = 2.92), while those with mild ISC were more likely to complete the program (z = −2.32).

Similarly, a one-way ANOVA examined adherence across levels of comparative self-criticism (CSC): mild (n = 165), moderate (n = 140), and severe (n = 150). The effect of CSC on completion rates was significant, F(2, 452) = 18.26, *p* < 0.001. Participants with moderate CSC completed significantly fewer modules (M = 6.89, SD = 2.35) compared to both the mild (M = 7.82, SD = 0.91; *p* < 0.001) and severe (M = 7.81, SD = 0.91; *p* < 0.001) groups, who did not differ from each other. A chi-square test confirmed a significant association between CSC levels and dropout, χ^2^(2) = 28.26, *p* < 0.001. Post hoc analysis showed that dropout was more likely among participants with moderate CSC (z = 3.63) and less likely among those with mild CSC (z = −2.70).

### 3.4. Main Results

#### 3.4.1. Missing Data

Among the participants who completed the intervention (N = 413), 110 (26.63%) did not complete the post-intervention questionnaires. Baseline comparisons revealed no significant differences between completers and non-completers across all measured variables, suggesting missing data were Missing Completely At Random (MCAR). A Little’s MCAR test was performed to confirm the randomness of missing data across post-intervention variables. The result was non-significant (χ^2^(81) = 35.96, *p* = 1.00), supporting the assumption that data were missing completely at random (MCAR). Therefore, all analyses were conducted on complete cases (n = 303). This approach, commonly recommended in nonrandomized designs with MCAR, ensures valid inferences while avoiding the assumptions required for imputation-based methods [35].

#### 3.4.2. Pre-Post Changes Controlling for Covariates

We hypothesized that different levels of self-criticism may differentially influence key therapeutic processes, including intervention outcomes. Therefore, a series of repeated-measures GLMs was conducted to examine changes in well-being, emotional awareness, rumination, worry, perfectionism, experiential avoidance, and assertiveness from pre- to post-intervention, while controlling for baseline levels of internalized self-criticism (ISC) and comparative self-criticism (CSC). This preliminary analysis aimed to determine whether self-criticism significantly influences these outcomes. If significant effects were observed, further analyses were planned to explore differences across subgroups characterized by mild, moderate, and severe levels of self-criticism.

As shown in Table 2, significant interactions between Time × ISC emerged for difficulty identifying feelings (TAS-20), personal standards and concern over mistakes (MPS), rumination (RRS), and psychological inflexibility (AAQ-II), suggesting that changes in these outcomes over time varied depending on participants’ initial levels of ISC. Moreover, significant interactions between Time × CSC emerged for concern over mistakes (MPS), psychological inflexibility (AAQ-II), and general assertiveness, frequency scale (SIB), suggesting that changes in these outcomes over time varied depending on participants’ initial levels of CSC.

#### 3.4.3. Differences Across Sub-Groups

Building on the previous findings (Table 2), which indicated that changes over time in difficulty identifying feelings, personal standards, concern over mistakes, rumination, psychological inflexibility, and assertiveness were influenced by baseline levels of self-criticism, we conducted further analyses using the categorizations previously presented in Table 1, which differentiated participants into mild, moderate, and severe levels of internalized self-criticism (ISC) and comparative self-criticism (CSC). The aim was to gain a better understanding of how different levels of self-criticism may affect intervention outcomes. Therefore, a series of repeated-measures ANOVAs was conducted, with Time (pre vs. post) as a within-subject factor and Group (Mild vs. Moderate vs. Severe ISC/CSC) as a between-subject factor. For outcomes showing a significant Time × Group interaction, Bonferroni post hoc tests were conducted to examine pre-post changes within each self-criticism group. ANOVA results are summarized in Table 3, and post hoc tests are detailed below. Please refer to the Supplementary Materials to view the graphics (“B–ANOVA and post hoc results”).

##### Difficulty Identifying Feelings

Following the significant interaction between time and internalized self-criticism subgroups (Table 3), post hoc analyses were conducted. Post hoc tests revealed significant pre-post reductions in all three groups (all *p* values < 0.001). The magnitude of change (Δ) differed across groups: the Mild group decreased by 1.98 points (SE = 0.41), the Moderate group by 1.98 points (SE = 0.48), and the Severe group by 3.53 points (SE = 0.47), suggesting greater improvements among participants with higher levels of internalized self-criticism.

##### Personal Standards (Perfectionism)

Given the significant interaction between time and internalized self-criticism subgroups (Table 3), post hoc analyses were conducted. Post hoc analyses suggested that the Severe group showed a significant decrease (*p* = 0.034), whereas the Mild and Moderate groups showed no significant change.

##### Concern over Mistakes Dimension (Perfectionism)

Considering the significant interaction between time and both internalized and comparative self-criticism subgroups (Table 3), post hoc analyses were conducted. For the internalized self-criticism groups, post hoc comparisons revealed a significant reduction only in the Severe self-criticism group (*p* < 0.001). For the comparative self-criticism groups, Bonferroni post hoc tests indicated that only participants in the Severe self-criticism group exhibited a significant decrease from pre- to post-intervention (*p* = 0.003).

##### Rumination

Following the significant interaction between time and internalized self-criticism subgroups (Table 3), post hoc analyses were conducted. Post hoc analyses suggested that only participants in the Severe self-criticism group exhibited a significant decrease from pre- to post-intervention (*p* < 0.001).

##### Psychological Inflexibility

Considering the significant interaction between time and both internalized and comparative self-criticism subgroups (Table 3), post hoc analyses were conducted. For the internalized self-criticism groups, significant pre-post reductions were found in both the Severe (*p* < 0.001) and Moderate (*p* = 0.012) groups, whereas the Mild group showed no significant change. The magnitude of change (Δ) differed across groups: the Moderate group decreased by 2.68 points (SE = 0.79), and the Severe group by 5.47 points (SE = 0.77), suggesting greater improvements among participants with higher levels of internalized self-criticism. For the comparative self-criticism groups, subgroup analyses did not reveal a significant Time × Group interaction for psychological inflexibility dimensions (F(2, 300) = 2.83; *p* = 0.06).

##### Assertiveness

Given the significant interaction between time and comparative self-criticism subgroups (Table 3), post hoc analyses were conducted. Bonferroni post hoc test suggested that only the Mild group showed a significant decrease (*p* < 0.001).

### 3.5. Working Alliance

It was hypothesized that higher levels of comparative self-criticism would be associated with a weaker working alliance, potentially due to participants’ negative perceptions of the relationship with the guide. First, Pearson correlation analysis was conducted to investigate the association between working alliance and self-criticism. Results showed no significant relationship between working alliance and internalized and comparative self-criticism (Table 4). Given the essence of association, no further analyses were conducted.

## 4. Discussion

The present study examined the role of self-criticism in adherence, working alliances, and treatment outcomes in NoiBene, a guided self-help intervention that promotes psychological well-being and prevents psychological distress among university students [16]. The results of the study partially confirmed the initial hypothesis and provided new insights.

Self-criticism appeared to play a role in both adherence and drop-out. Specifically, participants with moderate self-criticism showed lower adherence and higher dropout rates than those with mild or severe self-criticism. This pattern may be related to differences in motivation. Although only a trend toward statistical significance, students with severe comparative self-criticism more frequently reported participating in the intervention to address perceived problems rather than solely for self-understanding or self-improvement. This pattern suggests that their motivation to resolve specific difficulties may be stronger than that of individuals with moderate self-criticism, for whom perceived problems may feel less urgent. For the more severe self-critical group, the intervention may have been perceived as a crucial lifeline, enabling them to tolerate difficulties and remain engaged [4].

Regarding intervention outcomes, changes over time in difficulty identifying feelings, personal standards, concern over mistakes, rumination and psychological inflexibility, were influenced by baseline self-criticism. Subgroup analyses showed that participants with severe internalized and comparative self-criticism achieved the largest improvement across these domains. These findings align with emerging evidence indicating that high self-criticism can predict greater benefits in preventive and low-intensity interventions targeting maladaptive self-evaluation processes [11,12,13]. Notably, they also started from higher baseline scores than the mild and moderate groups, also exceeding population norms. This suggests that individuals with more severe self-criticism experience greater initial difficulties and therefore have more room for improvement, a phenomenon commonly described as a greater opportunity for therapeutic gain. Beyond this statistical consideration, it is possible that specific characteristics of the intervention contributed to these outcomes. First, individuals with high levels of self-criticism tend to be particularly concerned about mistakes [25]. The structured, skills-based format of NoiBene may have been especially well-suited to these individuals, as it offers clear, goal-oriented tasks that can be completed at one’s own pace, potentially reducing performance pressure and fear of failure. Second, the online format may have attenuated concerns related to social comparison and negative evaluation. Although the present study does not provide direct evidence that individuals high in self-criticism preferentially engage with digital interventions, it is possible to consider related psychological processes. For example, self-criticism has been consistently associated with higher levels of shame [36] and self-stigma [37], both of which have been linked to a greater preference for online mental health services [36,38]. In this context, the reduced interpersonal exposure inherent in digital formats may foster a perception of greater safety and lower threat, thereby facilitating engagement among individuals who are particularly sensitive to judgment. While these interpretations remain speculative, they underscore the potential role of intervention format in shaping treatment response and highlight the need for future research. Moreover, it is important to consider the interrelated nature of the psychological processes targeted in the intervention. A growing body of literature suggests that variables such as perfectionism, experiential avoidance, self-criticism, rumination, and worry form a mutually reinforcing network of negative self-evaluation and maladaptive coping processes [39,40,41]. These factors tend to co-occur and maintain one another over time, contributing to the persistence of psychological distress. From this perspective, the observed improvements may not be attributable to changes in a single process, but rather to the simultaneous targeting of multiple, interconnected mechanisms. By addressing this cluster of transdiagnostic factors, the intervention may have facilitated the disruption of self-reinforcing maladaptive cycles, thereby promoting broader and more robust psychological change.

Regarding working alliance, contrary to our hypothesis, it was not associated with comparative self-criticism. We had expected that individuals with high levels of comparative self-criticism, marked by chronic negative self-other comparisons and feelings of inferiority, would report a weaker alliance with their Tutor, potentially due to negative interpersonal expectations, heightened sensitivity to evaluation, or difficulty trusting others [5,17]. However, the absence of a significant correlation suggests that, within this guided self-help intervention, comparative self-criticism may not strongly impair participants’ perceptions of the collaborative relationship. One possible explanation lies in the nature of the intervention format. Unlike traditional face-to-face therapy, guided self-help interventions offer a more structured and less interpersonally demanding context, which may buffer the effects of interpersonal insecurity. For individuals high in comparative self-criticism, this semi-anonymous, less interactive format may reduce social-evaluative threat and mitigate concerns about being judged or ranked by the tutor, as it does for shy individuals [42] or for those with social anxiety [43].

### Limitations and Future Directions

This study has several limitations that should be acknowledged. First, participants voluntarily enrolled in the intervention, introducing a potential self-selection bias. Those who chose to participate may have been more motivated or receptive to self-help strategies than the broader population. This may have inflated adherence and perceived benefit rates. Second, although adherence was evaluated through quantitative indicators such as dropout rates and module completion, the study did not collect qualitative or process data to explore the reasons behind disengagement. As a result, we were unable to determine whether participants dropped out due to dissatisfaction with the intervention, a lack of time, perceived irrelevance, or worsening of their symptoms [44]. Future research should incorporate follow-up interviews or open-ended feedback to understand dropout dynamics better and inform more personalized strategies to enhance retention. Third, the working alliance was assessed only once at the end of the intervention, which may have captured a global, outcome-driven perception rather than the evolving, process-oriented quality of the relationship. Participants who experienced improvements may have retrospectively rated the alliance more positively, regardless of their initial relational tendencies or interpersonal difficulties. Previous research suggests that the impact of self-criticism on alliance may intensify over time [4], underscoring the need for multi-timepoint assessments to more accurately capture alliance development and possible ruptures. Moreover, this would not only allow for a more fine-grained understanding of how the alliance evolves over time but also has important clinical implications. Indeed, early indicators of weaker alliance may serve as markers of increased risk for dropout, thereby enabling the identification of participants who may benefit from targeted engagement strategies. In this sense, early assessment of the working alliance could represent a valuable tool not only for monitoring the therapeutic process, but also for preventing premature discontinuation of the intervention. Future research would benefit from assessing working alliance at multiple time points throughout the intervention and from incorporating both self-report and observer-rated perspectives. Moreover, it is important to note that participants who dropped out of the intervention did not complete the post-intervention questionnaires. It is possible that those individuals perceived the alliance as less positive, and their absence from the data may have biased the results toward an overestimation of alliance quality. Also, although the overall dropout rate of the intervention was low, indicating strong engagement, a notable proportion of participants who completed the entire intervention did not respond to the final assessment questionnaires. This presents a limitation for the study, as it reduced the available post-intervention data and may introduce bias into outcome analyses. However, this limitation should be interpreted with caution. From the perspective of the intervention itself, participants’ decision to complete the entire program despite not engaging in post-intervention research assessments may reflect a perception of usefulness or personal relevance. It is plausible that these individuals were primarily motivated by self-improvement rather than research participation, and that their non-response demonstrates a lack of interest in follow-up data collection rather than dissatisfaction with the intervention. Future studies may benefit from implementing strategies to increase post-assessment completion (e.g., reminders, incentives) and from collecting brief feedback on participants’ reasons for disengaging from the research component despite completing the intervention. A further limitation of the present study concerns the absence of follow-up assessments. Outcomes were evaluated only at post-intervention, preventing any conclusions regarding the stability and durability of the observed improvements over time. This aspect is particularly relevant in the context of self-criticism, which has been described as a relatively stable and trait-like construct [2], and may therefore be prone to relapse following the conclusion of an intervention. Future studies should incorporate follow-up measurements (e.g., at 3 and 6 months) to better assess the persistence of treatment effects and to identify potential patterns of relapse or sustained improvement. Lastly, the predominance of female participants in this study warrants further reflection. Evidence regarding gender differences in self-criticism is inconsistent. Some studies report higher average levels of dysfunctional self-criticism in women compared to men [45,46], whereas other research has failed to detect significant gender differences in dysfunctional self-criticism [47,48]. This inconsistency may be partly explained by sampling biases, as many studies on self-criticism rely on predominantly female samples [49], potentially limiting the ability to detect true gender differences. Men are historically underrepresented in clinical and research settings [50] and it is possible that men experience comparable levels of self-criticism that remain invisible in empirical data due to lower engagement with mental health services and lower participation in research. In this regard, consider that self-criticism is a known as associated with suicidality [51], a phenomenon that is significantly more prevalent among the male population [52]. In conclusion, the findings of the present study should be interpreted with caution, and further exploration of gender-specific patterns in self-criticism is clearly needed. When considering the generalizability of the findings, it is important to note that the sample was composed of university students, with a substantial proportion (approximately 37%) enrolled in psychology-related programs. However, the use of student samples does not necessarily imply limited generalizability per se, as such samples are widely used in psychological research. Concerns regarding convenience sampling are particularly relevant when the variables of interest show restricted variability within the sample. In the present study, the sample appeared relatively heterogeneous in terms of both demographic characteristics and key psychological variables. This variability may partially mitigate concerns regarding the generalizability of the findings. Nonetheless, caution is warranted when extending these results to populations with different demographic or clinical characteristics.

## 5. Conclusions

Overall, these findings challenge the notion that severe self-criticism is always a barrier to progress. They suggest that, in the context of tailored, structured, and non-threatening interventions, individuals with severe self-criticism can be exceptionally responsive. The findings from this study offer several practical implications for the development and delivery of preventive guided self-help interventions. First, the results highlight that individuals with severe self-criticism, often considered vulnerable to disengagement in traditional therapy, can still derive meaningful benefits from structured, low-intensity formats. This suggests that NoiBene represents not only an accessible but also an effective preventive tool for addressing transdiagnostic factors in subclinical populations, even in those individuals high in self-criticism. These results are particularly relevant, considering that major organizations, such as the World Health Organization, emphasize that intervening early, when risk factors are present but before psychopathology develops, can significantly improve mental well-being and reduce the likelihood of future disorders [53]. Therefore, guided self-help programs, such as NoiBene, align with international recommendations for scalable, low-intensity, and cost-effective strategies that can reach large populations [10]. In this way, NoiBene contributes to a stepped-care model [18] that promotes mental well-being before symptoms become clinical, supporting a public health approach to mental health.

The study also highlights that even within guided self-help interventions, self-criticism remains a significant factor in shaping adherence and dropout rates. Although internet-based interventions are widely recognized for their efficacy, they face important challenges, including high dropout rates [44] and limited improvement in specific subgroups [54]. By shedding light on how different forms and levels of self-criticism influence engagement, this study provides new insight for researchers and practitioners seeking to enhance digital intervention design. In particular, it emphasizes the importance of monitoring early warning signs of disengagement in individuals who are moderately self-critical and tailoring motivational or support strategies accordingly. Overall, by clarifying how self-criticism affects treatment engagement and outcomes, the findings underscore the importance of developing more personalized, responsive, and psychologically attuned approaches to preventive mental health care in the digital age.

## Figures and Tables

**Table 1:** Subgrouping into mild, moderate, and severe self-criticism.

	Internalized Self-Criticism (ISC)		Comparative Self-Criticism (CSC)	
	**n**	**M (SD)**	**n**	**M (SD)**
Mild	163	2.88 (0.58)	165	2.41 (0.41)
Moderate	150	4.34 (0.37)	140	3.31 (0.19)
Severe	142	5.69 (0.59)	150	4.40 (0.54)

**Table 2:** General Linear Model results: interactions with covariates.

			Time × ISC			Time × CSC		
	Pre	Post	F (df = 301)	*p*	η^2^	F (df = 301)	*p*	η^2^
Well-being (WEMWBS)	42.28 (7.29)	43.01 (9.90)	0.21	0.65		0.03	0.86	
Emotional awareness (TAS-20)								
Difficulty identifying feelings	15.36 (5.51)	12.90 (5.15)	8.46	0.004	0.03	2.99	0.08	
Difficulty describing feelings	13.95 (4.94)	12.19 (4.49)	0.99	0.32		0.87	0.35	
Perfectionism (MPS)								
Personal Standards	19.94 (5.88)	20.01 (5.11)	16.66	<0.001	0.05	1.73	0.19	
Concern over Mistakes and Doubts about Actions	37.01 (11.49)	35.36 (10.76)	18.42	<0.001	0.06	5.75	0.02	0.02
Rumination (RRS)	46.04 (10.67)	44.19 (11.32)	8.44	0.004	0.03	0.40	0.56	
Worry (PSWQ)	50.44 (13.94)	47.35 (13.06)	1.24	0.27		0.27	0.60	
Psychological inflexibility (AAQ-II)	32.38 (10.80)	29.31 (9.81)	15.09	<0.001	0.05	11.01	0.001	0.04
Assertiveness (SIB)								
Level of distress	122.52 (32.17)	113.81 (32.06)	1.83	0.18		0.49	0.49	
Frequency	162.77 (28.92)	152.79 (31.37)	2.08	0.15		8.56	0.004	0.03

**Table 3:** Subgroups analysis: ANOVAs results.

	Mild ISC		Moderate ISC		Severe ISC		Time × Group		
	Pre	Post	Pre	Post	Pre	Post	F (df = 300)	*p*	η^2^p
Difficulty identifying feelings	13.66 (5.08)	11.68 (4.59)	14.64 (4.46)	12.66 (4.44)	18.29 (5.86)	14.76 (5.94)	3.80	0.02	0.02
Personal Standards	16.90 (5.24)	17.94 (4.72)	20.28 (4.80)	20.54 (4.94)	23.65 (5.44)	22.25 (4.72)	8.28	<0.001	0.05
Concern over Mistakes and Doubts about Actions	28.77 (8.15)	29.57 (8.72)	37.37 (7.76)	35.70 (8.37)	47.60 (9.34)	42.71 (10.75)	11.82	<0.001	0.07
Rumination	40.68 (9.63)	40.57 (10.25)	45.69 (8.32)	44.44 (11.15)	53.48 (9.63)	48.76 (11.26)	6.83	0.001	0.04
Psychological inflexibility	27.23 (8.33)	25.70 (8.79)	32.28 (8.85)	29.60 (8.36)	39.29 (11.63)	33.83 (10.51)	7.47	0.0007	0.05
	**Mild CSC**		**Moderate CSC**		**Severe CSC**		**Time × Group**		
	**Pre**	**Post**	**Pre**	**Post**	**Pre**	**Post**	**F** **(df = 300)**	***p***	**η^2^p**
Concern over Mistakes and Doubts about Actions	30.31 (8.57)	30.09 (9.30)	36.12 (9.20)	34.29 (8.40)	45.41 (10.75)	42.23 (10.32)	3.21	0.04	0.02
General assertiveness-Frequency scale	176.46 (28.53)	160.44 (36.74)	158.68 (25.34)	151.38 (26.26)	150.34 (25.41)	145.16 (26.25)	4.86	0.008	0.03

Note: ISC, internalized self-criticism; CSC, comparative self-criticism.

**Table 4:** Pearson correlational analysis between working alliance and self-criticism.

	1	2	3	4
1. Internalized self-criticism	—			
2. Comparative self-criticism	0.516 **	—		
3. Bond	0.028	−0.070	—	
4. Task and goal agreement	0.058	−0.005	0.533 **	—

** *p* < 0.001.

## Data Availability

Group-level information about the data is available from the corresponding author on reasonable request.

## Supplementary Materials

The following supporting information can be downloaded at: https://www.mdpi.com/article/10.3390/healthcare14081107/s1, A—NoiBene intervention; B—ANOVA and post-hoc results.

## Abbreviations

The following abbreviations are used in this manuscript:

ISC	Internalized self-criticism
CSC	Comparative self-criticism
