# Supplementary material for: Self-Criticism in Preventive Guided Self-Help Interventions: Greater Gains or Greater Risks? Its Effect on Adherence, Treatment Success, and Working Alliance
Source: Healthcare (Basel). 2026 Apr 21;14(8):1107. doi: 10.3390/healthcare14081107 (PMC13116946; doi:10.3390/healthcare14081107)
Supplement: Supplementary file 1 [file healthcare-14-01107-s001.zip › healthcare-4219551-supplementary.pdf]

SUPPLEMENTARY MATERIALS

A - NoiBene intervention

Module	Aims, contents, and exercises
1. Commitment and Motivation	The concept of well-being is explained according to Positive Psychology theories. Commitment and motivation are elicited in students by asking them to schedule a timetable of future access to the platform and to set personal goals they mean to reach with the intervention.
2. Self-awareness	Psychoeducation about emotions, basic individual needs, and values is proposed as a first step. Afterward, to help students become aware of their emotions and needs, they are asked to use a personal diary inspired by ABC technique. Lastly, some exercises are proposed to help students identify their values system.
3. Psychological Bugs	The module begins with introducing the concept of Psychological bugs (e.g., mental processes and attitudes contributing to emotional suffering). To identify students' vulnerabilities, they have to complete a series of exercises related to control strategies, such as thought suppression, avoidance, and distraction.
3.1. Repetitive thinking	Psychoeducation about rumination, worry, and self-criticism is proposed. Successively, students are asked to use a personal diary to monitor their repetitive thinking behavior, and to challenge such thoughts, they are encouraged to follow some techniques derived from Self-compassion therapy and Rumination-focused therapy.
3.2. Perfectionism	The module begins with psychoeducation about different forms of perfectionism. Later, students are asked to complete exercises to identify personal high standards and the positive and negative aspects of being a perfectionist. Then, students are encouraged to follow some techniques to reduce perfectionistic thoughts and behavior.
3.3. Avoidance	After psychoeducation about avoidance and psychological inflexibility, students are asked to use a personal diary inspired by ABC technique, focusing on the situations, thoughts, and emotions they tend to avoid. Students are then encouraged to follow some techniques to challenge avoidance.
4. Social Skills	This module aims to improve basic social competencies such as active listening, assertiveness, and gratitude to foster positive relationships. First, psychoeducation about active listening and communicative styles is provided. Moreover, students are asked to complete some exercises and self-monitoring tools to identify their predominant communicative style. Then, according to the Nonviolent Communication model, the various steps to develop assertive and empathic communication are presented. Some monitoring tools are proposed to understand the operating modes fully. Lastly, after some theoretical contents about gratitude, exercises such as the gratitude diary and the gratitude letter are proposed.
5. Self-realization	The module includes a series of contents and exercises to help students set specific, attainable, and realistic goals. Students are especially encouraged

	to set goals following their values (previously exposed in the Self-awareness module). The last part of the module is related to the study method. Different study methods are introduced after proposing a self-monitoring tool to identify any related difficulties, and students are helped to identify the best one that fits their needs and competencies. The module concludes by introducing different mnemonics (e.g., Loci Method)

B – ANOVA and post-hoc results

In every graphic: * p < .05; ** p < .01; *** p < .001; Vertical bars denote +/- standard errors

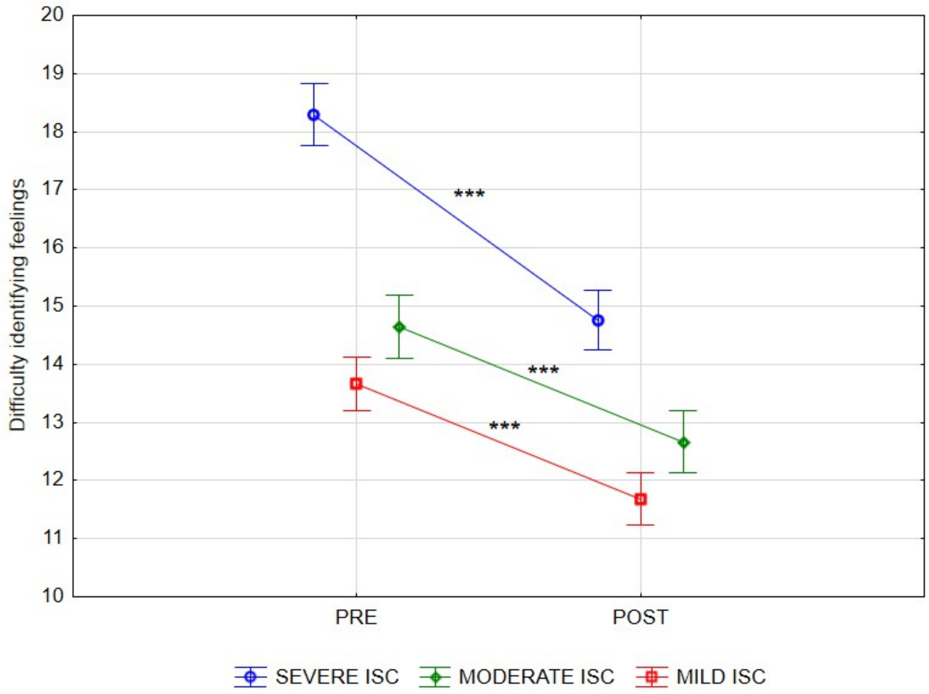

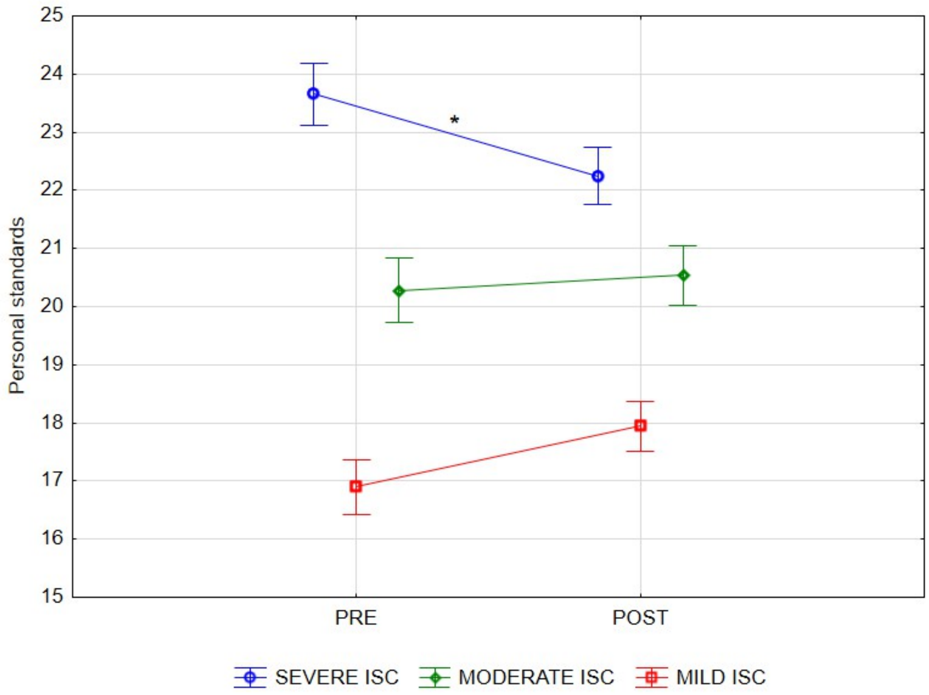

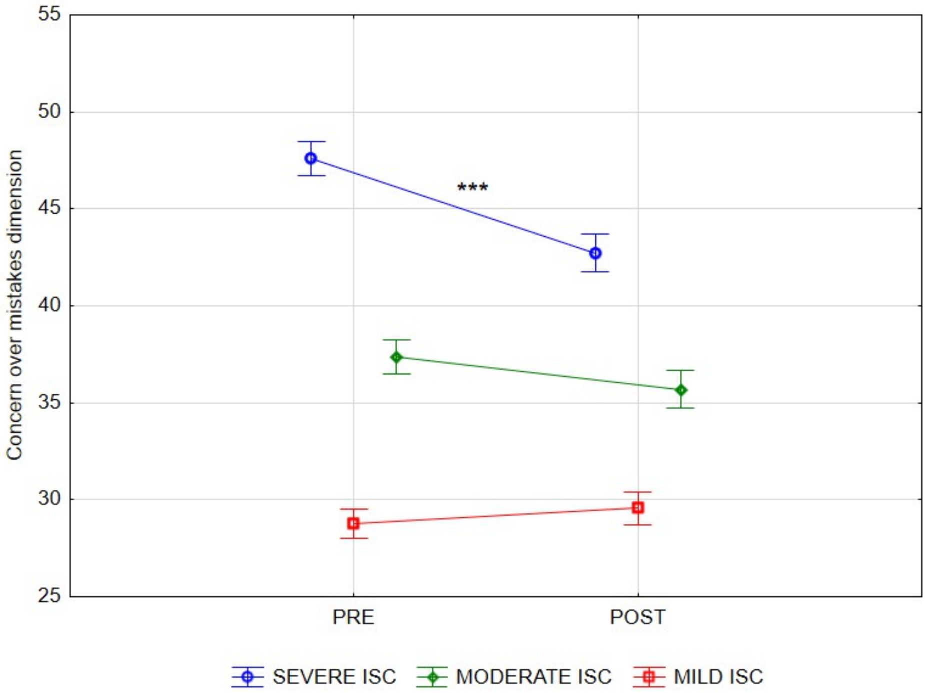

55
50
45
40
35
30
25
Concern over mistakes dimension
***
PRE
POST
SEVERE ISC
MODERATE ISC
MILD ISC

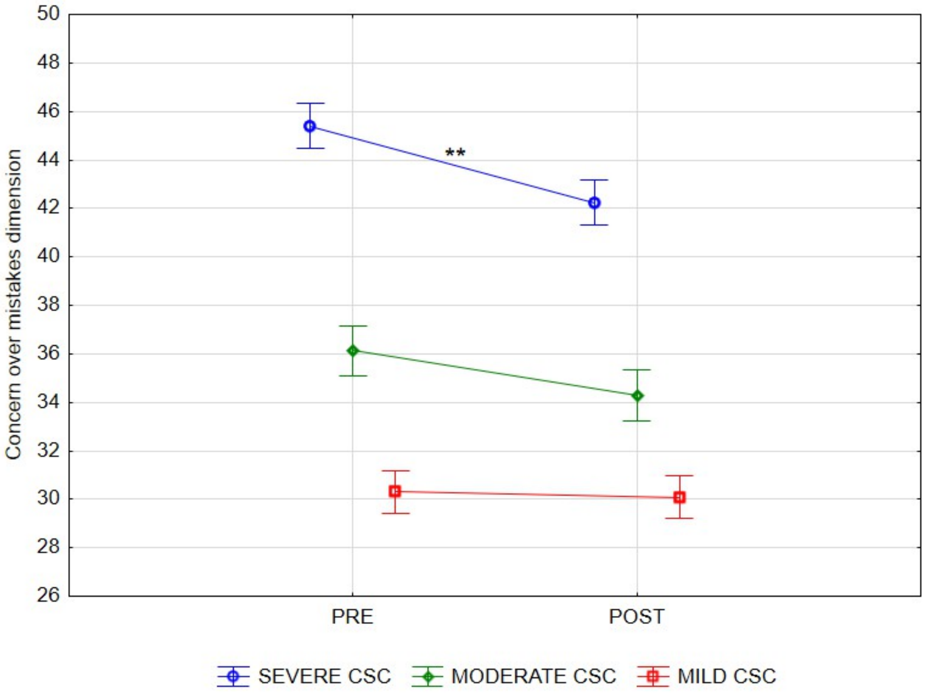

50
48
46
44
42
40
38
36
34
32
30
28
26
Concern over mistakes dimension
**
PRE
POST
SEVERE CSC
MODERATE CSC
MILD CSC

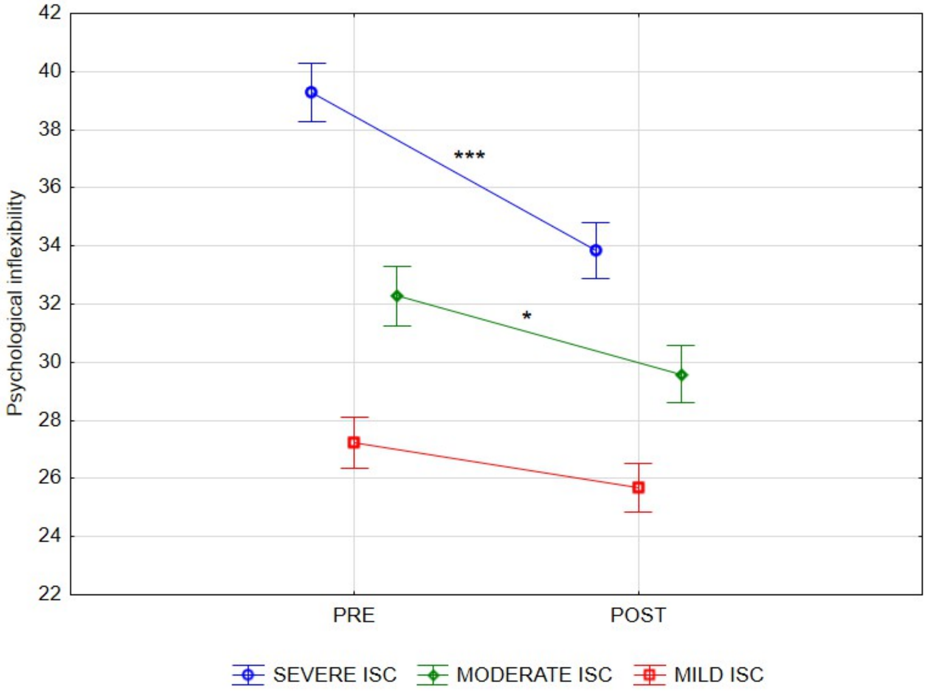
42
40
38
36
34
32
30
28
26
24
22
Psychological inflexbility
***
*
PRE
POST
SEVERE ISC
MODERATE ISC
MILD ISC

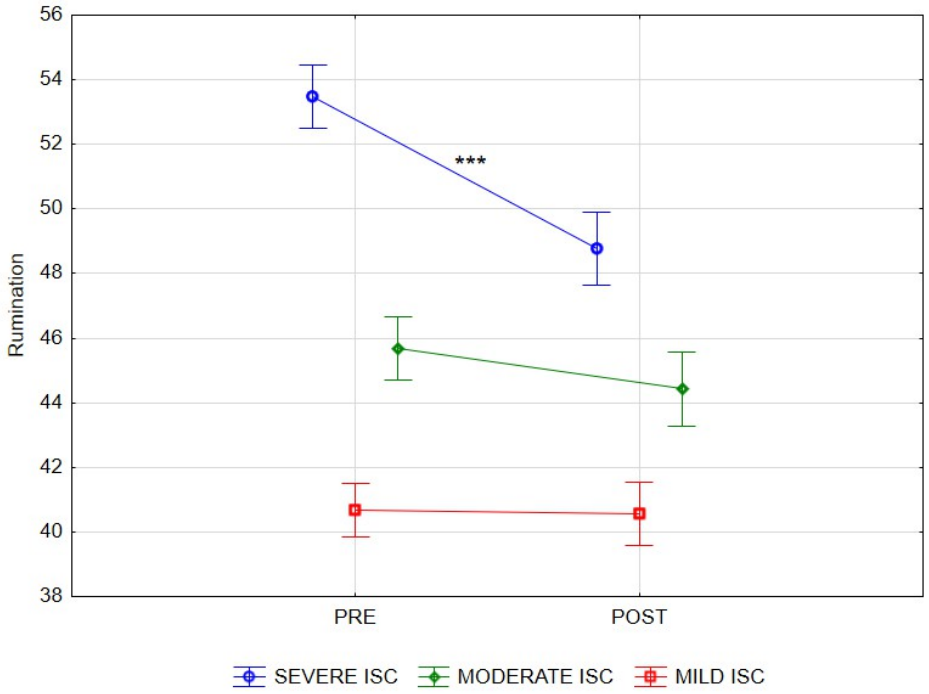
56
54
52
50
48
46
44
42
40
38
Rumination
***
PRE
POST
SEVERE ISC
MODERATE ISC
MILD ISC
